# Radiobiology Contributions and Perspectives in Hadron Therapy, With a Focus on Carbon Ions: Report From the Workshop Hadron Therapy for Life, Caen, March 2025

**DOI:** 10.1016/j.ijpt.2025.101289

**Published:** 2025-11-26

**Authors:** Siamak Haghdoost, Juliette Thariat, Iuliana Toma-Dasu, Benjamin Frey, Claire Rodriguez-Lafrasse, Samuel Valable, Carine Laurent, Dinu Stefan, Francois Chevalier, Angelica Facoetti, Ivana Dokic, Takashi Shimokawa, Piero Fossati, Walter Tinganelli, Christophe Rochais, Gabriel Gaubert, Jean-Louis Habrand

**Affiliations:** 1 Université Caen Normandie, Univ Rouen Normandie, Normandie Univ, ABTE UR4651, Caen, France; 2 Advanced Resource Center for HADrontherapy in Europe (ARCHADE), Caen, France; 3 Département de Radiothérapie, Centre François Baclesse, Caen, France; 4 Oncology Pathology Department, Karolinska Institutet, Stockholm, Sweden; 5 Medical Radiation Physics, Stockholm University, Stockholm, Sweden; 6 Translational Radiobiology, Department of Radiation Oncology, Universitätsklinikum Erlangen, Friedrich-Alexander-Universität Erlangen-Nürnberg, Erlangen, Germany; 7 Laboratory of Cellular and Molecular Radiobiology, UMR CNRS5822/IP2I, Lyon-Sud Medical School, Univ Lyon, Lyon 1 University, Oullins, France; 8 Department of Biochemistry and Molecular Biology, Groupement Hospitalier Sud, Hospices Civils de Lyon, Pierre-Bénite, France; 9 Université Caen Normandie, CNRS, Normandie Univ, ISTCT UMR6030, Caen, France; 10 SAPHYN (SAnté et PHYsique Nucléaire), Caen, France; 11 Université Caen Normandie, ENSICAEN, CNRS, CEA, Normandie Univ, CIMAP UMR6252, Caen, France; 12 Radiobiology Unit, CNAO National Center for Oncological Hadrontherapy, Pavia, Italy; 13 Division of Molecular and Translational Radiation Oncology, Heidelberg Ion-Beam Therapy Center (HIT), Heidelberg Faculty of Medicine (MFHD), Heidelberg University Hospital (UKHD), German Cancer Research Center (DKFZ) and National Center for Tumor Diseases (NCT), Heidelberg, Germany; 14 Radiation Effect Research Group, Department of Accelerator and Medical Physics, Institute for Quantum Medical Science, National Institutes for Quantum Science and Technology (QST), Chiba, Japan; 15 Department of Radiation Oncology, MedAustron Ion Therapy Center, Wiener Neustadt, Austria; 16 Biophysics Department, GSI Helmholtzzentrum für Schwerionenforschung, Darmstadt, Germany; 17 Université Caen Normandie, Normandie Univ, CERMN UR4258, Caen, France; 18 CYCLHAD, Multi-ion Hadron Therapy Center, Caen, France

**Keywords:** Radiobiology, Radioresistance, Molecular radiobiology, Hypoxia, Immunotherapy

## Abstract

The “Hadrontherapy for Life” symposium in Caen, France, highlighted that a new era of radiobiology is fundamental for advancing particle therapy to the next level. A radiobiology capable of integrating molecular biology and omics technologies is needed to deeply analyze treatment responses and underlying mechanisms.

Key challenges discussed at the symposium included tumor hypoxia, which remains only partially mitigated by high-LET radiation, and the specificity of carbon ions, or more broadly, high-LET particles, considered as “new drugs” capable of providing systemic benefits beyond local tumor control, including their potential to promote immunogenicity. Moreover, emerging modalities, such as Ultra High Dose Rate irradiation and spatial fractionated beams, were also discussed, with consensus that all require dedicated and coordinated radiobiological investigations.

Infrastructure presentations highlighted the international capabilities of leading centers in Europe and Asia, emphasizing the importance of integrating radiobiology into clinical programs, advancing multi-ion experimentation, and adopting innovative experimental models, such as organoids and/or 3D cell cultures. Participants also stressed the need for greater access to animal experimentation facilities, which are essential for accelerating progress in the field. Furthermore, the meeting underscored translational endpoints such as biomarker development, a hot topic in current radiotherapy. The C400 accelerator enables Caen to incorporate radiobiology from its very inception, establishing a European hub for collaborative research. Round-table discussions emphasized the importance of harmonized protocols, dedicated in vivo irradiation rooms, international training programs with exchange of students and researchers, and comprehensive patient biobanking.

In summary, the symposium reinforced the essential role of radiobiology in advancing hadron therapy (HT), providing strategic directions for translational research, infrastructure development, and international collaborations to accelerate personalized and effective particle therapy.

## Introduction

The “Hadrontherapy for Life” symposium was held in March 2025 in Caen, France, marking a significant milestone with the completion of the C400 accelerator, an advanced platform dedicated to both research and clinical applications in hadron therapy (HT). Over the past decades, radiobiology has evolved alongside the clinical implementation of particle therapy. Early carbon ion treatments at HIMAC-QST (Japan) and GSI (Germany) were supported by basic biological insights but with limited robust translational frameworks. The establishment of dedicated clinical centers such as HIT (Germany), CNAO (Italy), and MedAustron (Austria) positioned radiobiology as a key element of treatment planning, patient selection, and therapeutic innovation.^1^ Today, with more than 100 proton centers and nearly 15 carbon ion facilities worldwide, the need for rigorous radiobiological frameworks is greater than ever.

Classical radiobiology, based on low-LET radiation such as photons and electrons, established the foundations of dose–response modeling, DNA damage and repair kinetics, and cell survival through the linear–quadratic model. Photon irradiation produces sparsely ionizing tracks, inducing mainly indirect damage via water radiolysis, leading predominantly to simple DNA lesions that are efficiently processed by base excision and homologous recombination repair pathways. In contrast, hadron beams, comprising neutrons and charged particles such as protons and carbon ions, exhibit dense ionization along their tracks, yielding highly clustered and complex DNA double-strand breaks with limited reparability. This microdosimetric heterogeneity explains the increased relative biological effectiveness observed with HT. Importantly, the transition from photon-based to hadron-based paradigms challenges classical assumptions regarding oxygen enhancement ratio (OER), dose-rate effects, and sublethal damage repair. High-LET irradiation can partially overcome hypoxia-induced radioresistance and diminish the protective impact of efficient DNA repair phenotypes, yet residual resistance mechanisms persist in certain molecular subtypes, suggesting LET-dependent modulation of signaling, chromatin architecture, and checkpoint control. Consequently, the extrapolation of photon-based radiobiological models to HT requires a refined understanding of track structure biology and a systematic reassessment of the determinants of radioresistance within high-LET contexts.

Several challenges highlight this need: (i) tumor hypoxia, which continues to drive resistance despite the partial oxygen independence of high-LET radiation; (ii) molecular specificity, with carbon ions engaging distinct DNA repair and signaling pathways compared to photons; (iii) the integration of HT with immunotherapy, where the immunogenic potential of hadrons remains insufficiently understood; (iv) the emergence of novel modalities such as ultra-high dose rate irradiation, spatial fractionated beams, and multi-ion therapy, each requiring radiobiological validation; and (v) the presence of cancer stem cells for which response to HT needs to be further evaluated.

In parallel, international infrastructures have matured to address these challenges, with centers such as HIT, GSI, CNAO, MedAustron, and HIMAC-QST developing integrated biology platforms and translational pipelines. The commissioning of the C400 accelerator in Caen provides a new opportunity to embed radiobiology from the outset in both clinical and research programs, in alignment with European initiatives such as ENLIGHT and EU-funded consortia that promote collaboration, training, and harmonization of standards.

Against this backdrop, the *Hadrontherapy for Life* symposium convened international experts to review the state of the art, identify gaps, and propose strategic directions for radiobiology in HT. The Radiobiology Session focused on three central themes: hypoxia, molecular specificity, and combined HT with immunotherapy, supplemented by presentations of major laboratory infrastructures worldwide. A concluding round table distilled expert recommendations for future development, particularly in the context of Caen and the forthcoming commissioning of C400.

The present paper synthesizes these discussions, offering a structured overview of current knowledge, infrastructures, and strategic priorities in hadron radiobiology. By integrating scientific highlights with expert consensus, it aims to define a roadmap for future research that will reinforce the biological foundations of HT and accelerate its clinical translation.

## Scientific highlights

### Hypoxia and tumor resistance

A key unresolved question in the field is whether HT can overcome hypoxia. Although carbon ions exhibit a lower OER than photons, the effect remains modest at clinically relevant LET ranges. In spread-out Bragg peaks, OER values of 2-3 persist, indicating that hypoxia-related resistance is not fully eliminated. Interpretation of outcomes is further complicated by the frequent comparison of hypofractionated carbon-ion regimens with conventionally fractionated photon regimens, since hypofractionation alone may mitigate hypoxia effects regardless of radiation quality.^2^ Combining of hypoxia-targeted imaging (eg, FMISO- or FAZA-PET) or MRI-based readout and hypoxia-modifying agents such as nimorazole could enhance the efficacy of HT. Advances in tumor oxygenation monitoring and the development of hypoxia-specific radiosensitizers also offer new opportunities. Addressing hypoxia requires multimodal strategies, combining HT with imaging, pharmacological modulation, and optimized fractionation schedules.

### Molecular specificity of carbon ion irradiation

Carbon ion irradiation induces unique molecular and spatial signatures. Along the ion track, clustered ROS production generates highly complex DNA damages that are difficult to repair, an effect sometimes called the “Bomber effect,” reflecting efficient tumor cell eradication. In contrast, the low proportion of ROS outside the tracks is not able to trigger the cellular mechanisms of defence and proliferation*.* This “Stealth effect” contributes to the sparing of normal tissue and may underlie the favorable therapeutic index of carbon ion therapy.^3^

At the signaling level, carbon irradiation has been reported to suppress pathways associated with invasion and metastasis, including MEK, JNK, STAT3, and Akt/mTOR. If validated in clinical settings, this could position carbon ion therapy not only as a local treatment modality but also as a potential systemic anti-metastatic strategy. A particularly relevant dimension of molecular specificity concerns the response of cancer stem cells (CSCs), a subpopulation characterized by enhanced self-renewal, quiescence, and intrinsic radioresistance. Conventional photon irradiation often spares cancer stem cells (CSCs) and hypoxic niches due to their efficient DNA repair mechanisms, high antioxidant capacity, and activation of pro-survival pathways such as Notch, Wnt/β-catenin, and Hedgehog. In contrast, carbon ion radiation induces complex, irreparable DNA damage and oxidative stress that can override these defense mechanisms. Preclinical data suggest that carbon ions more effectively ablate CSCs and disrupt their niche signaling, thereby reducing tumor repopulation and metastatic potential. Despite their increased susceptibility to high-LET damage, subsets of CSCs may retain resistance through genotype-dependent repair or signaling adaptations, warranting comprehensive molecular and functional analyses of LET-specific CSC responses.^4^, ^5^

Incorporating molecular endpoints into clinical trials is important. Genomic, proteomic, and metabolomic analyses should be systematically integrated into HT patient cohorts to capture treatment-specific biological effects, including the modulation of CSC populations and signaling networks that drive recurrence and resistance.

### HT and immunotherapy

Ionizing irradiation not only induces DNA damage in cells but can also result in the release of cytosolic DNA fragments that activate innate immune pathways such as the cGAS–STING pathway. Whether this process translates into durable systemic antitumor immunity depends strongly on the immunogenicity of the cell death as well as the micro- and macro-environment of the tumor. Compared with photons, protons and carbon ions may generate distinct “radiation immune fingerprints.” Carbon ion irradiation, for example, produces clustered DNA damage that can favor immunogenic cell death. In contrast, certain proton-based combinations (eg, with nanoparticles) may induce cell death without effective immune priming. These observations highlight the complexity of radiation–immune interactions.

From a translational perspective, combining HT with immune checkpoint inhibitors (ICIs) represents a promising strategy, but clinical data remain scarce. Preclinical in vivo models are needed to capture systemic and abscopal effects that cannot be replicated in vitro and also allowing to differentiate between ballistic and biological effects between photons and hadrons. Future clinical protocols should integrate immunomonitoring and biomarker-driven patient selection, as variability in immune response will likely determine which patients benefit most from HT–ICI combinations.^6^ It is important to note that before large-scale studies are conducted with immunotherapy, the effects of hadron radiation also need to be examined more closely in patient studies and highlighted from a molecular and immunological perspective. This is mainly to clearly demonstrate an additive effect with immunotherapy.

### Infrastructure presentations

Progress in radiobiology critically depends on access to high-quality irradiation facilities. While conventional X-ray and gamma sources are sufficient for in vitro studies, investigations of charged particles require specialized infrastructures capable of delivering protons or heavy ions under clinically relevant conditions. Over the past 2 decades, several centers worldwide have established radiobiology laboratories adjacent to particle accelerators, fostering translational environments that linking physics, biology, and medicine.

At the Caen Symposium, 6 major centers presented their capacities and future directions: CNAO (Italy), HIT (Germany), GSI (Germany), MedAustron (Austria), HIMAC-QST (Japan), and the local laboratories in Caen (France). Together, these institutions illustrate the diverse strategies for integrating radiobiology into HT, ranging from 2D/3D cell culture studies and in vivo animal platforms to patient biobanking.

Cross-cutting themes emerged across facilities:

• Clinical integration: radiobiology programs are closely linked with patient treatment, ensuring bidirectional translation between bench and bedside.
• Expansion to multi-ion capability: in addition to protons and carbon ions, helium, oxygen, and heavier ions are increasingly available, broadening the scope of biological questions.
• Animal irradiation platforms with easy access: several centers are developing or upgrading in vivo facilities, acknowledging their central role in bridging preclinical and clinical research.
• Translational endpoints: research now extends beyond fundamental mechanisms (DNA repair, chromatin remodeling, oxidative stress) to biomarkers, immune modulation, and normal tissue toxicity.
• International access: most centers provide external user access through European programs, national initiatives, or institutional collaborations.

CNAO (Italy) integrates radiobiology directly into its clinical operations, currently supporting 2D and 3D cell irradiation and planning small-animal facilities. The center is expanding its research infrastructure to include Boron Neutron Capture Therapy, leveraging accelerator-based neutron sources to explore new therapeutic options for resistant tumors. In parallel, CNAO is advancing the development of helium, oxygen, and lithium beams, with research priorities in tumor resistance, multimodality approaches, and HT–immunotherapy synergies. Through the HITRIplus program (European Union’s Horizon 2020 grant agreement No 101008548), CNAO offers beam time to external users, reinforcing its role as a European hub for advanced radiotherapy research (https://www.hitriplus.eu).

HIT (Germany) is hospital-based proton-, helium-, and carbon ion center, which combines patient care with translational research. Beyond cell and animal models, it emphasizes tumoroid and organoid models for studying different cancer diseases. HIT is pioneering FLASH irradiation with higher LET-particles, ion mini-beam radiotherapy and helium program, exploring paradigms that may improve therapeutic selectivity.

GSI (Germany)**,** historically the first clinical carbon ion center, now operates exclusively for research. Its accelerators provide ions up to uranium, uniquely positioning GSI for both radiobiology and space research. Facilities include full cell and animal labs, imaging platforms, and dedicated groups on DNA repair, translational radiobiology, space radiobiology, hypoxia, cytogenetics, immune modulation, and FLASH effects. Beam allocation is overseen by a scientific advisory committee, ensuring high-quality output.

MedAustron (Austria) sustains a strong non-clinical research program supported by dedicated infrastructure and long-term funding. Offering protons, carbon, and helium ions, it prioritizes translational studies on reoxygenation, high-LET boost strategies, immune modulation, and DNA repair inhibitors, serving as a model for sustainable integration of radiobiology into clinical centers.

HIMAC-QST (Japan) remains one of the world’s most versatile heavy ion facilities, with multiple accelerators delivering a wide ion spectrum (He to Fe). Over one-third of its radiobiology projects are international, supported by flexible access schemes. Research spans DNA repair, cardiovascular effects, radiation protection, and emerging modalities such as FLASH, GRID, and mini-beams.

Caen (France) hosts three laboratories, ISTCT, CIMAP, and ABTE, with access to proton, carbon ion, and X-ray platforms. Proton irradiations are performed at the CYCLHAD facility using the Proteus One system, and carbon ion irradiations at GANIL on the iRiA-CIRIL platform, which provides ion beams from carbon to uranium under the oversight of a scientific advisory committee. X-ray irradiations are conducted at the CYCERON facility. Research in Caen focuses on normal tissue toxicity, resistant tumors, combined modality approaches, and carbon FLASH therapy. While the lack of hadron-based animal facilities remains a limitation, CYCERON’s advanced animal care and imaging platform offers unique opportunities for in vivo follow-up. Its proximity to the forthcoming C400 accelerator is expected to enable fully translational research and further consolidate Caen’s leadership in radiobiology and particle therapy.

In summary, the infrastructure session underscored both the strengths and disparities of global radiobiology capacities. Together, these centers form the backbone of experimental HT, offering complementary expertise, ion species, and access models. Coordinated use of these infrastructures, facilitated by European networks and international partnerships, will be essential for addressing key biological and clinical questions in the coming decade.

### Round table recommendations

The expert panel synthesized following strategic priorities:

1. Education & Training, develop Erasmus-type programs, mobility networks (ENLIGHT).
2. In vivo irradiation, urgent need for small animal irradiation at Caen.
3. Funding & Access, EU/international grants, cost-sharing models for beam time.
4. Patient Biobank, systematic collection of blood/tissue from hadron-treated patients.
5. Clinical Alignment, design studies addressing fractionation, hypoxia, and immunotherapy combinations.

## Discussion

The session confirmed radiobiology’s pivotal role in advancing HT, while underscoring several unresolved challenges. Hypoxia remains only partially mitigated by high-LET radiation, requiring multimodal approaches that combine imaging, pharmacological modulation, and optimized fractionation. Similarly, the immunogenicity of particle irradiation is highly context-dependent and requires deeper translational research for effective clinical integration. At the molecular level, the unique biological signatures of carbon ions suggest potential systemic benefits beyond local tumor control, opening promising avenues for future therapeutic development.

Comparisons between international infrastructures highlighted distinct strategic axes: Japan prioritizes multi-ion biology and long-term clinical follow-up, Germany focuses on translational pipelines and novel modalities such as FLASH and mini-beam irradiation, while Italy and Austria illustrate strong integration of radiobiology into clinical programs. In France, the commissioning of the C400 accelerator represents a unique opportunity to embed radiobiology from the outset. Looking ahead, key research frontiers include FLASH HT and spatial radiation; where they may differentially spare normal tissues; organoid models as bridges between cell-based and in vivo studies; and multi-omics platforms that could enable predictive, patient-tailored applications of HT.

### Future perspectives

The commissioning of the C400 accelerator positions Caen as an important hub for European HT research. Radiobiology must be tightly integrated into the C400 program to maximize its clinical impact.

The upcoming PTCOG 64 congress (2026, Normandy) will coincide with the initiation of beam production from C400. This provides a unique opportunity to showcase European leadership and establish Caen among the reference centers for basic and translational heavy-ion biology.

Looking forward, 3 imperatives stand out:

1. Integration of radiobiology into clinical protocols, biomarker-driven trials, immune monitoring, and hypoxia imaging.
2. International collaboration, coordinated EU grants, shared infrastructures, and harmonized protocols.
3. Sustainability, long-term funding, training programs, and patient-centered translational pipelines.

## Conclusions

Radiobiology is fundamental to fully realizing the potential of HT. The Caen Symposium underscored a global consensus on the necessity of robust translational infrastructures, strengthened international collaborations, and close clinical integration. Together, these efforts will accelerate progress toward more effective and personalized cancer treatments.

## CRediT authorship contributions statement

Conceptualization, Data curation, Methodology, Validation, Review and Editing; all authors. Funding acquisition JLH, JT, GG, SH. Original draft Writing SH, JLH and JT.

## Declaration of Conflicts of Interest

The authors declare that they have no known competing financial interests or personal relationships that could have appeared to influence the work reported in this paper.
